# Interference and Mechanism of Dill Seed Essential Oil and Contribution of Carvone and Limonene in Preventing Sclerotinia Rot of Rapeseed

**DOI:** 10.1371/journal.pone.0131733

**Published:** 2015-07-02

**Authors:** Bingxin Ma, Xiaoquan Ban, Bo Huang, Jingsheng He, Jun Tian, Hong Zeng, Yuxin Chen, Youwei Wang

**Affiliations:** Institute of TCM and Natural Products, School of Pharmaceutical Sciences, Wuhan University, Wuhan, P.R. China; Fujian Agriculture and Forestry University, CHINA

## Abstract

This study aimed to evaluate the inhibitory effects of dill (*Anethum graveolens* L.) seed essential oil against *Sclerotinia sclerotiorum* and its mechanism of action. The antifungal activities of the two main constituents, namely carvone and limonene, were also measured. Mycelial growth and sclerotial germination were thoroughly inhibited by dill seed essential oil at the 1.00 μL/mL under contact condition and 0.125μL/mL air under vapor condition. Carvone also contributed more than limonene in inhibiting the growth of *S*. *sclerotiorum*. Carvone and limonene synergistically inhibited the growth of the fungus. *In vivo* experiments, the essential oil remarkably suppressed *S*. *sclerotiorum*, and considerable morphological alterations were observed in the hyphae and sclerotia. Inhibition of ergosterol synthesis, malate dehydrogenase, succinate dehydrogenase activities, and external medium acidification were investigated to elucidate the antifungal mechanism of the essential oil. The seed essential oil of *A*. *graveolens* can be extensively used in agriculture for preventing the oilseed crops fungal disease.

## Introduction

*Sclerotinia sclerotiorum* (Lib.) de Bary is a soil-borne plant pathogen that has caused significant damage on most vegetables, particularly oil crops [1,2]. This fungus is homothallic and does not produce asexual spores [3]. Diseases caused by *S*. *sclerotiorum* have traditionally been difficult to control. Recently, naturally developed substances have increased because of the negative effects of misuse and overuse of synthetic chemical fungicides and fertilizers; these disadvantages include fungicide resistance, toxic residues, and negative environmental effects [4, 5]. Consequently, the use of naturally derived compounds, such as essential oils and plant extracts, as potential fungicides has greatly increased [6]. Rapeseed (*Brassica napus* L.) is grown for the production of animal feed, vegetable oil, biodiesel and lubricant-derived products. Rapeseed is frequently infected with *Sclerotinia sclerotiorum* during crop cultivation, thereby causing considerable economic losses [4, 7]. This pathogen has usually caused 20% to 50% rapeseed yield loss in China [8].

Dill (*Anethum graveolens* L.) belongs to the Apiaceae family. In China, dill is primarily cultivated in the Xinjiang Uyghur Autonomous Region. Dill is also used for its pharmacological properties, such as antibacterial and antifungal activities [9–12]. Carvone and limonene, as volatile components, have been extensively used for their numerous pharmacological activities. Carvone has also been used in agriculture for its antifungal activity [13]. Limonene, which is derived from α-terpinyl cation, is a constituent extensively found in essential oils and can also be used as an antifungal agent [13, 14].

In the current study, we determined the interference and mechanism of dill seed essential oil in preventing *S*. *sclerotiorum* infection. The effects of carvone, limonene, and a mixture of the two were also measured. These two chemicals were used to evaluate their contributions to the activity of dill seed essential oil. Carvone and limonene were mixed on the basis of their proportion in dill seed essential oil to evaluate their interaction. We systematically proceeded with the study to elucidate epiphyte-disease-control mechanisms and further extend the practical applications of dill as a novel fungicide in agriculture.

## Materials and Methods

### Plant material and fungal pathogen

Dill seeds were purchased from the local market in Urumuqit, Xinjiang Uyghur Autonomous Region of China and identified by Prof. Qing Wang (Wuhan Botanical Garden, Chinese Academy of Sciences). A voucher specimen of the samples (No. 581) was deposited to the Institute of Traditional Chinese Medicine and Natural Products, School of Pharmaceutical Sciences of Wuhan University. Air-dried dill seeds (200 g) were hydrodistilled for 3 h by using a Clevenger-type apparatus [15]. Carvone and Limonene were purchased from Sigma (Sigma-Aldrich Trading Co., Ltd., China). *Sclerotinia sclerotiorum* strains were provided and identified by the College of Plant Science and Technology of the Huazhong Agricultural University and deposited in the Culture Collection of State Key Laboratory of Agricultural Microbiology (No. 041196).

### GC-MS analysis

Gas chromatography (GC)–mass spectrometry (MS) analysis was performed. Diluted dill seed essential oil was injected into GC-MS (QP-2010, Shimadzu Co., Kyoto, Japan) and separated on Rtx-5MS column (30 m length, 0.25 mm internal diameter, and 0.25 μm thickness) by using helium as the carrier gas. The GC oven was operated at an initial temperature of 60°C for 2 min. The temperature was programmed to increase at 10°C∙min^−1^ up to a final temperature of 280°C, which was held for 5 min before cooling. Both injector and detector temperatures were at 280°C. The mass spectrometer was operated in 70 eV EI mode with scanning from 45 amu to 450 amu for 0.5 s, and the ion source was set at 200°C. Essential oil components were identified by comparing their retention times and mass fragmentation patterns with those stored in the MS database through NIST05.LIB and NIST05s.LIB (National Institute of Standards and Technology).

### Interference of the essential oil *in vitro*

#### Effect of the essential oil on mycelial growth of *S*. *sclerotorium*

Antifungal activity toward mycelial growth of *S*. *sclerotiorum* was assessed in both contact and vapor phases [16,17]. For the contact phase effect, different concentrations of dill seed essential oil were dissolved in sterile Tween 20-water solution (0.10% v/v) to obtain final concentrations of 0.00, 0.25, 0.50, 0.75, and 1.00 μL/mL. The samples were then transferred to sterile 90 mm glass Petri plates (20 mL/plate). At the center of each Petri plate, an agar disc with 6 mm diameter was placed.

For the vapor phase, glass Petri plates were filled with 20 mL of the medium, and one disc was placed on potato dextrose agar in each plate as previously described. Each Petri plate was diluted by adding 1 mL of 0.00, 4.00, 6.00, 8.00, or 10.00 μL/mL dill seed essential oil to obtain concentrations of 0.000, 0.050, 0.075, 0.100, and 0.125 μL/mL of oil in air. The Petri plates were then sealed with parafilm and inoculated as previously described. Three replicates were prepared for each concentration. Mycelial growth inhibition (%) = [(*d*_*c*_ − *d*_t_)/*d*_*c*_] × 100, where *d*_*c*_ and *d*_t_ represent mycelial growth diameters in the control and treatment Petri plates, respectively.

#### Effect of dill seed essential oil on sclerotial viability in sand treatment

The effect on the viability of *S*. *sclerotiorum* was measured using the method of Soylu et al. [18] with slight modifications. For the contact effect, 20 mL of steam-sterilized natural sand at the bottom dish of a Petri plate was mixed with different concentrations of dill seed essential oil (0.00, 0.25, 0.50, 0.75, and 1.00 μL/mL). Ten sclerotia were buried under 0.5 cm of sand.

For the vapor effect, sterile filter papers with different concentrations of dill seed essential oil were placed on the medium-free cover to obtain final concentrations (0.000, 0.050, 0.075, 0.100, and 0.125 μL/mL) of essential oil in air. Ten sclerotia were placed on the sand surface. The plates of both contact and vapor effects were sealed with parafilm then incubated at 20°C for 8 d. The germination rate was obtained using the number of sclerotia showing mycelial growth compared with that of the control group. Each treatment was prepared in triplicate.

#### Contribution of carvone and limonene on antifungal activities *in vitro*

To evaluate the contributions of carvone, limonene, or a mixture of these compounds, we assessed the activities of mycelial growth inhibition and sclerotial viability of *S*. *sclerotiorum* at both contact and vapor phases in accordance with the above methods. For mycelial growth at contact phase, the concentrations of carvone and limonene were set based on their proportions (limonene: 32.63% and carvone: 41.51%) in dill seed essential oil (Table 1). Thus, the concentrations of limonene were 0.00, 0.08, 0.16, 0.24, and 0.33 μL/mL, and those of carvone were 0.00, 0.10, 0.20, 0.31, and 0.42 μL/mL. The concentrations of the mixture of carvone and limonene were the combined corresponding concentrations of limonene and carvone as follows: 0.00, 0.18, 0.36, 0.55, and 0.75 μL/mL. Thus, in vapor phase, the concentrations of the mixture in air were 0.000, 0.037, 0.055, 0.073, and 0.093 μL/mL of air; those of limonene in air were 0.000, 0.016, 0.024, 0.033, and 0.041 μL/mL; and those of carvone in air were 0.000, 0.021, 0.031, 0.042, and 0.052 μL/mL. For sclerotial viability, the same serial concentrations were used to evaluate the contributions.

**Table 1:** Chemical composition of essential oil isolated by hydrodistillation from dill seed.

Peak No.	R.I.	Compound	Relative concentration (%)
1	902	α- Thujene	tr
2	948	α- Pinene	tr
3	958	β- Myrcene	tr
4	964	β- Phellandrene	tr
5	969	α- Phellandrene	0.51
6	998	γ- Terpinene	0.10
7	1018	Limonene	32.63
8	1031	*trans*- Limonene oxide	tr
9	1042	*p*-Cymene	0.18
10	1103	Anethofuran	tr
11	1121	*trans*-Fenchone	tr
12	1179	*cis*-Dihydrocarvone	2.56
13	1179	*trans*-Dihydrocarvone	3.35
14	1190	Carvone	41.51
15	1196	Dihydrocarveol	0.22
16	1196	Neodihydrocarveol	0.33
17	1206	*cis*-Carveol	0.24
18	1507	Caryophyllene oxide	tr
19	1516	Myristicin	0.40
20	1568	Asarone	tr
21	1705	Apiol	16.79
		Total identified	98.82

R.I., retention index on the Rtx-5MS column. tr (trace), relative content < 0.10%.

### Interference of dill seed essential oil *in vivo*

#### Effect of dill seed essential oil against *Sclerotinia sclerotiorum* in detached oilseed rape (*Brassica napus* L.) leaves

Oilseed rape seeds were planted outdoors in plastic pots in August, 2013. Five weeks after planting, two leaves with similar shapes were detached from plants that yielded an average of four to six leaves. The leaves were placed on a filter paper in Petri dishes and placed in an incubator. *In vivo* control of *S*. *sclerotiorum* infection of the leaves with different concentrations of dill seed essential oil (0.00, 0.25, 0.50, 0.75, and 1.00 μL/mL; 0.10% Tween 20-water solution) was accomplished by two methods. First, the leaves were inoculated with a 6 mm-diameter mycelial agar plug. The plug was cut from the growing margins of a 4 d old *S*. *sclerotiorum* culture 12 h before 5 mL of the oil was separately sprayed on each of the two leaves per Petri dish. The second *in vivo* control method followed the same procedure but was performed 12 h after the treatment with dill seed essential oil. Two leaves per Petri dish represented a replicate, and the experiment was performed in triplicate. Petri dishes were sealed with parafilm then incubated at 20°C under fluorescent light [19].

#### Effect of essential oil against *Sclerotinia sclerotiorum* in potted oilseed rape plants

The tested oilseed rape seedlings showed an average of 8 to 15 leaves and were cultivated under controllable greenhouse conditions at a temperature of 20°C to 25°C conducted in six replicates. The antifungal efficacy of the test samples was evaluated after 5 d as a percentage of inhibition calculated by the following formula: percent inhibition (%) = [(*A* − *B*)/*A*] × 100, where *A* and *B* represent the disease areas on the untreated and treated plants, respectively.

### Antifungal mechanism of the essential oil

#### Effects of the essential oil on hyphal and sclerotial morphology

Mycelia and sclerotia were treated for 3 and 10 d with 0.50 and 1.00 μL/mL of (contact phase) dill seed essential oil. Subsequently, mycelial discs (1 cm diameter) and sclerotia were fixed with 2.50% glutaraldehyde in 0.10 M phosphate buffer (pH 7.2) for 2 h at room temperature [16]. They were then washed with the phosphate buffer twice. Afterward, the samples were dehydrated thrice in a gradient ethanol series (70%, 80%, 90%, and 100%) until dried. Double-sided carbon tape was used for installing the fixed material. Gold/palladium was used for coating in a high vacuum chamber for 150 s at 9 mA. The samples were examined, and digital images were obtained by Hitachi S-570 SEM (Hitachi Ltd., Tokyo, Japan) at an accelerating voltage of 20 kV.

#### Determination of ergosterol amount

Ergosterol content of *S*. *sclerotiorum* was measured as described by Czaczyk and Jun [20]. Agar discs (6 mm diameter) were inoculated in potato dextrose broth medium containing 0, 0.25, 0.5, and 0.75 μL/mL of dill seed essential oil for 6 d at 20°C. Mycelia were harvested and washed thrice with distilled water after incubation. Up to 10 mL of 25% alcoholic potassium hydroxide solution was added to each sample and mixed by vortex for 10 min. The mixture was then incubated at 85°C for 2 h. Sterols were extracted by adding a mixture of sterile distilled water (2 mL) and n-heptane (5 mL). The mixture was homogenized by vortex for 10 min. The heptane layer was stored at −20°C for 24 h. Before analysis, a 1 mL aliquot of the sterol extract was diluted twofold in ethanol. Scanned spectrophotometry (UV-1700, Shimadzu, Tokyo, Japan) was used to analyze the n-heptane layer between 230 and 300 nm. The ergosterol content was calculated using our previous equations [20].

% ergosterol + % 24(28) dehydroergosterol = [(A282/290) × *F*]/pellet weight;

% 24(28) dehydroergosterol = (A230/518 × *F*)/pellet weight;

% ergosterol = [(A282/290) × *F*]/pellet weight − (A230/518 × *F*)/pellet weight.

In these formulae, *F* is the factor for dilution in ethanol; 290 and 518 are the constant values; A282 is the absorbance at 282nm; A230 is the absorbance at 230 nm.

#### Preparations of mitochondria

The mitochondria of *S*. *sclerotiorum* were isolated as previously described with slight modifications [20, 21]. Mycelium (2 g) was collected using the previously described methods. The mycelium was washed twice with 10 mL of sterile buffer containing 50 mM Tris (pH 7.5), 2 mM EDTA (ethylene diamine tetraacetic acid), 1 mM PMSF (phenylmethanesulfonyl fluoride), then resuspended in the same buffer. The mixture was transferred into centrifuge tubes then ultrasonically treated five times to break the cytoderm. Glucose (2%) was supplemented after the cytoderm was broken. The homogenate was centrifuged at 2000 × *g* for 5 min to remove mycelial fragments and conidia. Supernatant was transferred into a new centrifuge tube and centrifuged at 10000 × *g* for 30 min. Mitochondria were resuspended in buffer for use.

#### Measurement of malate dehydrogenase and succinate dehydrogenase activities in mitochondria

Malate dehydrogenase (MDH) and succinate dehydrogenase (SDH) activities in essential oil-treated fungal cells were detected using a Micro-ATPase Assay Kit obtained from the Institute of Biological Engineering of Nanjing Jiancheng (Nanjing, China) in accordance with the manufacturer’s protocol.

#### Determination of external medium acidification

Acidification of external medium was measured as described by Jun [20]. Mycelia (1.0 g) were suspended in 40 mL of 50 mM KCl and refrigerated at 4°C overnight. Dill seed essential oil was added to the suspension to acquire final concentrations of 0, 0.25, 0.5, and 0.75 μL/mL, and the treatment was conducted for 10 min. After filtration, the mycelium was treated with 20 mL of 10% glucose solution. The pH value was measured every 10 min.

### Statistical analysis

The results were reported as mean ± SD. To determine statistical significance, we conducted ANOVA using SPSS (version 20.0). Least significant difference (*p* < 0.05) was applied to separate mean values.

## Results

### Chemical composition of the essential oil

The oil obtained from the dill seed was yellowish with strong characteristic aroma, and the yield was 1.80%. GC-MS analysis of dill seed essential oil identified 23 different components that represented 98.82% of the total oil. The identified compounds are listed in Table 1 according to their elution order on the Rtx-5MS column. The oil contained a complex mixture of olefinic hydrocarbons and monoterpenes, along with some essential phytochemicals. The major components detected in oil were carvone (41.51%), limonene (32.63%), apiol (16.79%), *trans*-dihydrocarvone (3.35%), *cis*-dihydrocarvone (2.56%), 2,5-diethylphenol (0.62%), α-phellandrene (0.51%), myristicin (0.40%), neodihydrocarveol (0.33%), *p*-cymene (0.18%), dihydrocarveol (0.22%), *cis*-carveol (0.24%), and γ-terpinene (0.10%).

### Interference of essential oils *in vitro*

**Fig 1:**
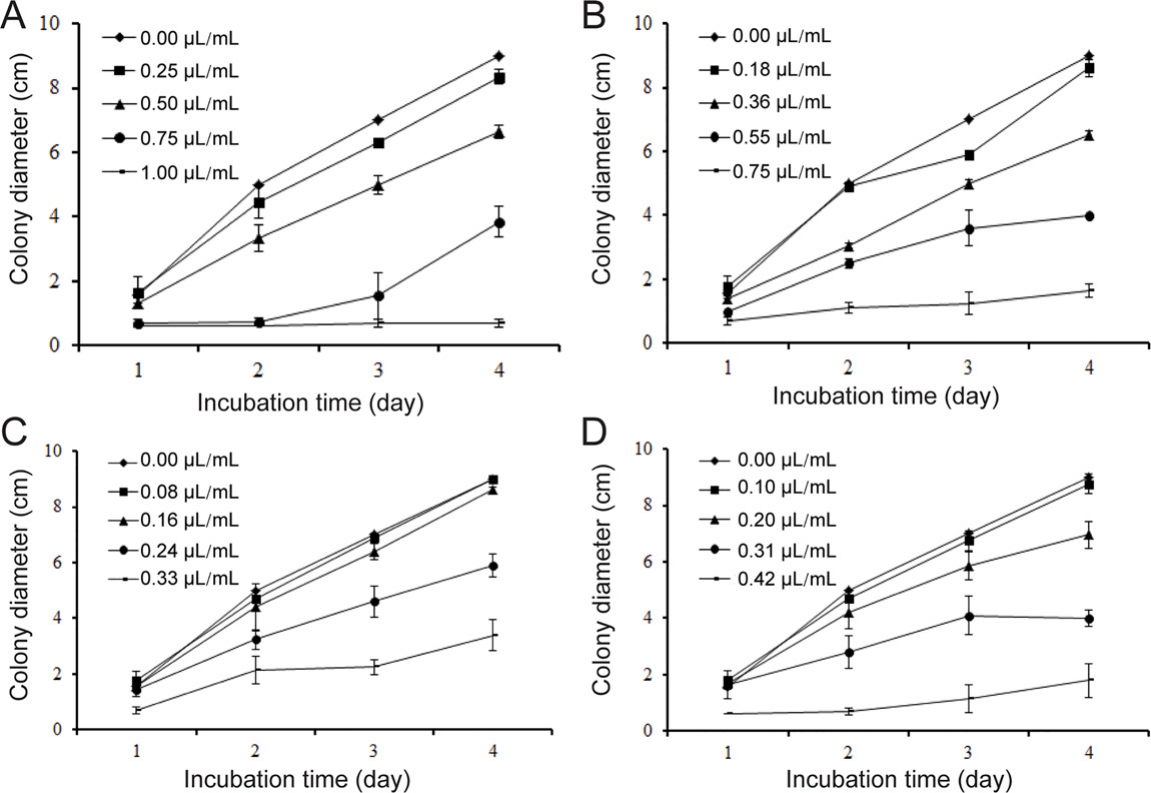
Effects of the samples at contact phase on colony diameter (cm) growth of *S*. *sclerotiorum*. (A) The oil, (B) mixture of carvone and limonene, (C) limonene, (D) carvone. Values are means (*n* = 3) ± standard deviations.

**Fig 2:**
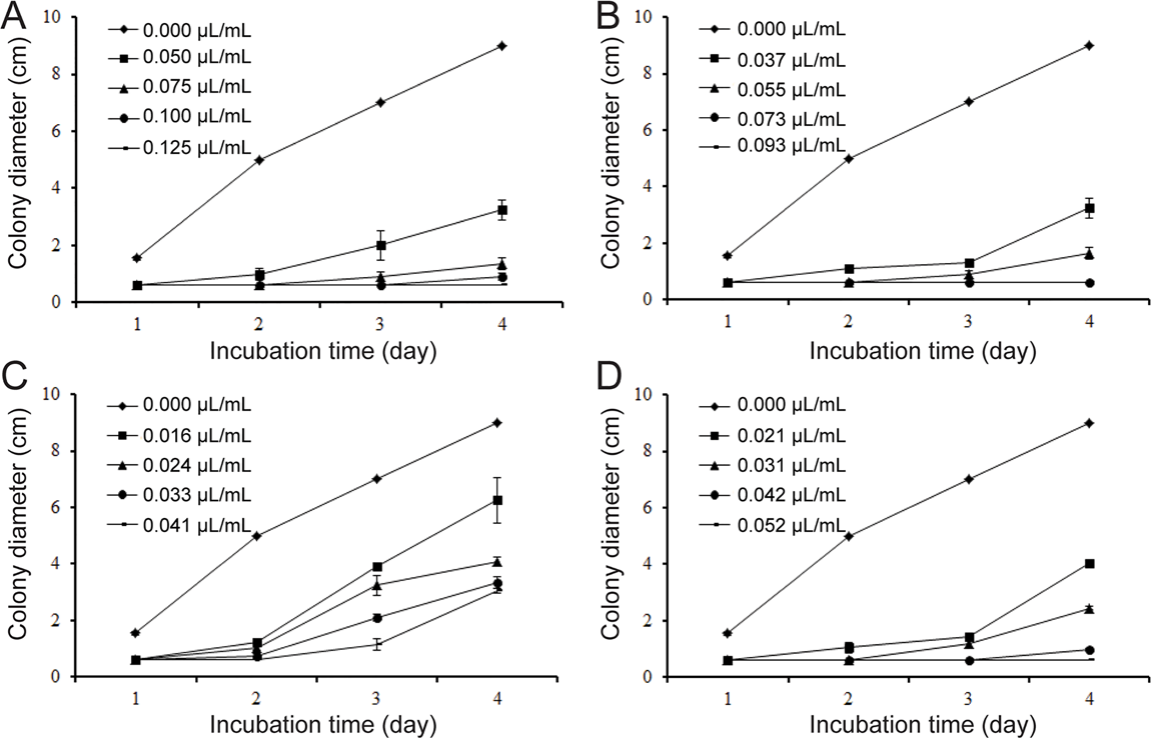
Effects of the samples at vapor phase on colony diameter (cm) growth of *S*. *sclerotiorum*. (A) The oil, (B) mixture of carvone and limonene, (C) limonene, (D) carvone. Values are means (*n* = 3) ± standard deviations.

The contact phase effects of different concentrations of the essential oil, carvone, limonene and the mixture of carvone and limonene on mycelial growth of *S*. *sclerotiorum in vitro* are shown in Fig 1A, 1B, 1C and 1D. Dill seed essential oil (1.00 μL/mL) completely inhibited fungal growth after 4 d of incubation. The mixture exhibited better activity than both carvone and limonene (Fig 1B). Carvone contributed more than limonene to the inhibitory activity of essential oil (Fig 1C and 1D). The contribution of carvone and limonene was normalized by inhibition percent. The average contributions of carvone and limonene were 77.35% and 19.16%, respectively. The contribution of the mixture was 82.47%. The vapor phase effects of dill seed essential oil, the mixture of carvone and limonene, carvone, and limonene were greater than their contact inhibitory effects on mycelial growth. Results of the vapor phase effects are shown in Fig 2. In the vapor phase test at 0.125 μL/mL essential oil, 0.093 μL/mL the mixture, and 0.052 μL/mL carvone in air, fungal development was completely inhibited after 4 d of incubation. Furthermore, 0.041 μL/mL limonene can delay the growth for 2 d. The inhibitory percent of the mixture is also similar to that of essential oil.

**Fig 3:**
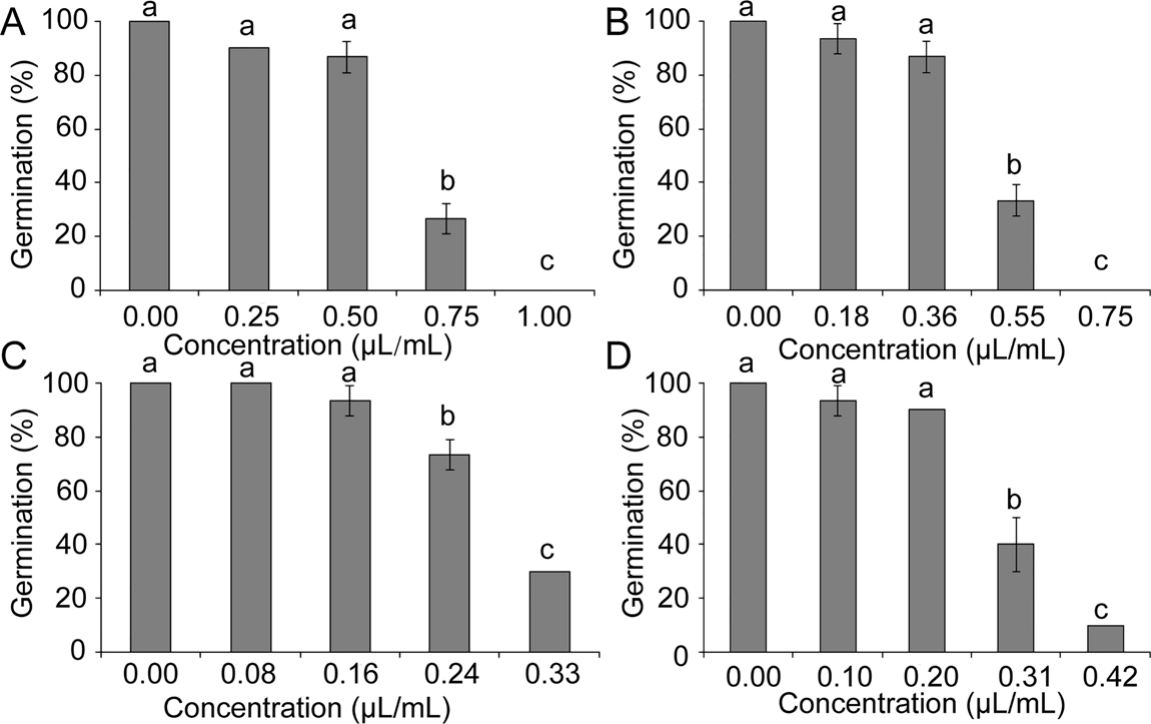
Effects of the samples at contact phase on sclerotial germination of *S*. *sclerotiorum*. (A) The oil, (B) mixture of carvone and limonene, (C) limonene, (D) carvone. Significant differences (*p* < 0.05) between means are indicated by the letters above histogram bars. Values are means (*n* = 3) ± standard deviations.

**Fig 4:**
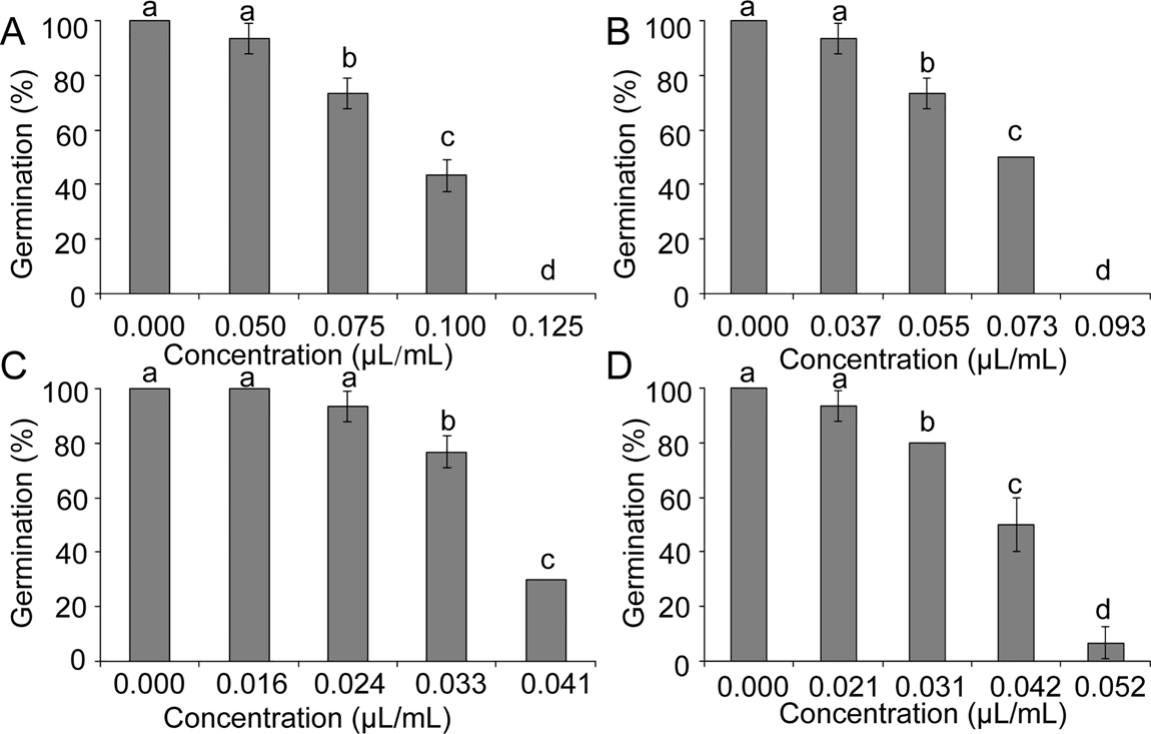
Effects of the samples at vapor phase on sclerotial germination of *S*. *sclerotiorum*. (A) The oil, (B) mixture of carvone and limonene, (C) limonene, (D) carvone. Significant differences (*p* <0.05) between means are indicated by the letters above histogram bars. Values are means (*n* = 3) ± standard deviations.

The contact and vapor phase effects on sclerotial viability were evaluated after 8 d of incubation in sand amended with different concentrations of essential oils (Figs 3 and 4). At high concentrations, essential oil and the mixture of carvone and limonene can completely inhibit sclerotial germination for 8 d (Fig 3A and 3B and Fig 4A and 4B). The sclerotia treated with limonene also demonstrated higher contribution than those treated with carvone in inhibition of germination (Fig 3C and 3D and Fig 4C and 4D).

### Interference of the essential oil *in vivo*

The treatment of detached oilseed rape leaves with different concentrations of the essential oil, before and after inoculation with mycelial agar plug of *S*. *sclerotiorum*, resulted in significant differences among samples in suppressing lesion development. Compared with the untreated controls, treatment of the leaves with essential oils at 12 h before inoculation was visibly more effective in suppressing lesion development than the post-inoculation treatment (Table 2), except at low oil concentrations. Furthermore, dill seed essential oil (0.50 μL/mL) completely suppressed lesion development. The oil exhibited a dose-dependent antifungal activity on *S*. *sclerotiorum* at concentrations tested after inoculation with mycelial plugs. The mean leaf lesion diameter ranged from 8.67 mm to 17.83 mm.

**Table 2:** Mean lesion size following direct inoculation of detached oilseed rape leaves with mycelial agar plugs 12 h before and after treatment with concentration range of dill seed essential oil.

Material	Concentration	Mean lesion diameter (mm)	
		Before inoculation	After inoculation
Dill seed essential oil	0.25 μL/mL	12.08 ± 0.49 ^a^	17.83 ± 2.08 ^a^
	0.50 μL/mL	0.00 ± 0.00 ^b^	13.00 ± 0.54 ^b^
	0.75 μL/mL	0.00 ± 0.00 ^b^	10.91 ± 1.11 ^c^
	1.00 μL/mL	0.00 ± 0.00 ^b^	8.67 ± 0.51 ^d^
Tween 20	0.10%	30.25 ± 1.92 ^c^	30.42 ± 1.69 ^e^
Carbendazol	1.00 mg/mL	28.42 ± 2.08 ^c^	26.67 ± 2.40 ^e^

Values are mean (*n* = 3) ± standard deviations. Values followed by same letters in a column are insignificantly different (*p* > 0.05).

**Fig 5:**
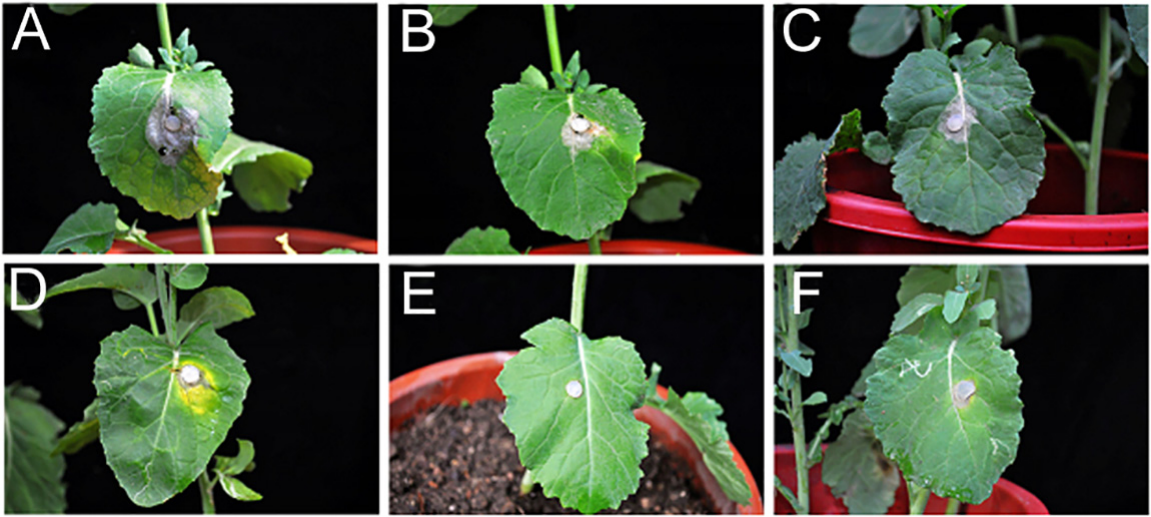
Efficacy of the oil against *S*. *sclerotiorum* in potted plants. (A) Control; (B)–(E) Treated with the oil (1.25, 2.50, 5.00, and 10.00 μL/mL); (F) Treated with carbendazol (1.00 mg/mL).

In potted plant tests, 1.25 μL/mL to 10.00 μL/mL of the oil inhibited *Sclerotinia* disease development 7 d after inoculation with *S*. *sclerotiorum*. Furthermore, the average reduction in disease was 40.78% to 100.00% compared with that of the control (Table 3). The interference of dill seed essential oil was remarkable at the highest concentration compared with carbendazol. By contrast, a moderate interferential effect was the characteristic feature of dill seed essential oil at low concentrations (Fig 5).

**Table 3:** *In vivo* antifungal activity of dill seed essential oil against plant pathogenic fungus of *S*. *sclerotiorum* on greenhouse-grown oilseed rape plants.

Material	Concentration	Disease suppression efficacy (%)
Dill seed essential oil	1.25 μL/mL	40.78 ± 3.46 ^a^
	2.50 μL/mL	45.73±7.65 ^a^
	5.00 μL/mL	65.01± 6.11 ^b^
	10.00 μL/mL	100.00 ± 0.00 ^c^
Tween 20	0.10%	0.00 ± 0.00 ^d^
Carbendazol	1.00 mg/mL	59.89 ± 5.98 ^b^

Values are mean (*n* = 6) ± standard deviations. Values followed by same letters in a column are insignificantly different (*p* > 0.05).

### Mechanism of the essential oil

**Fig 6:**
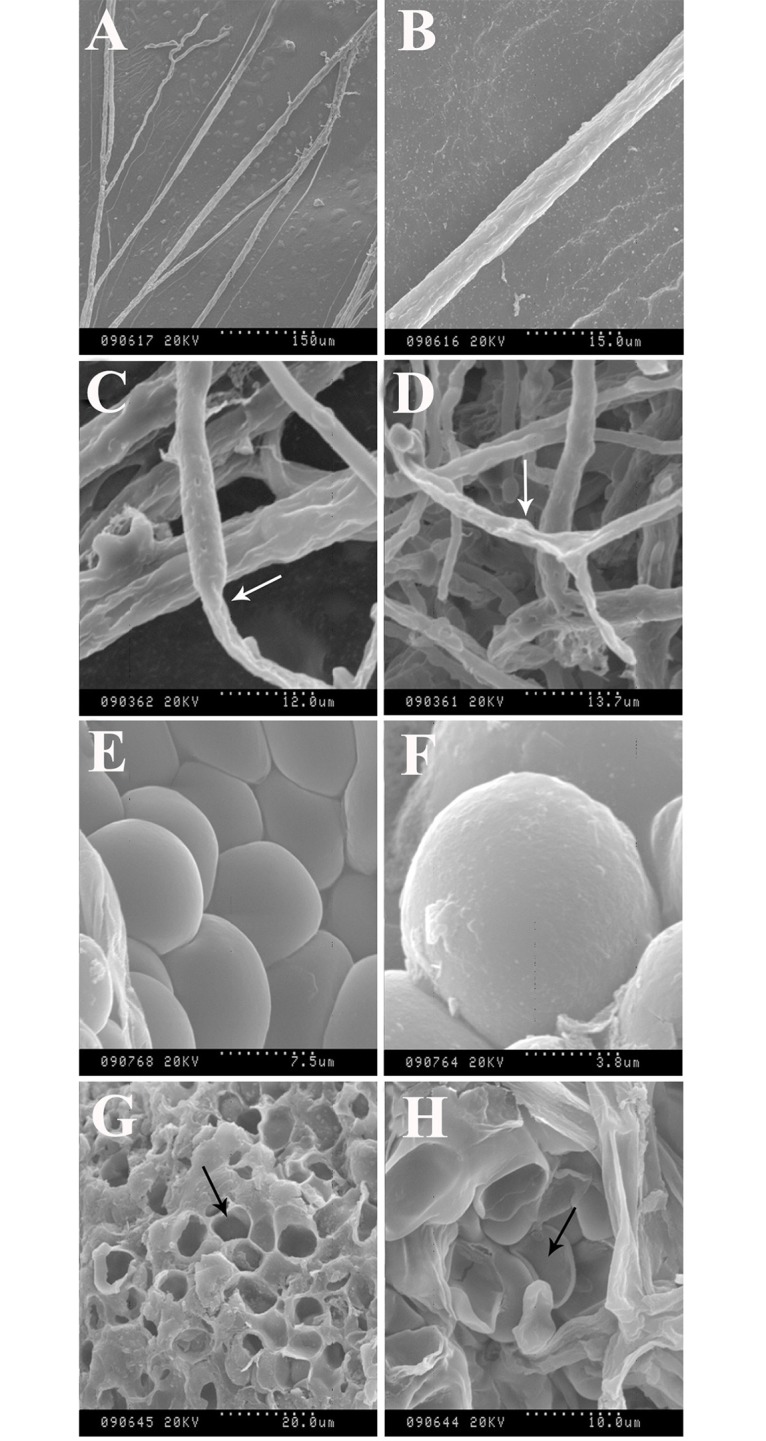
Scanning electron microscopy illustrated effects of the oil on microstructure surface of *S*. *sclerotiorum*. Control (A, B, E, and F). Effects of essential oil on hyphal morphology (C and D). Effects of essential oil on surfaces of sclerotia and rind globular cells inside the sclerotium (G and H).

The effects of the essential oil on mycelia and sclerotia of *S*. *sclerotiorum* were examined by SEM (Fig 6). The presence of essential oils in the culture medium resulted in a different morphology of *S*. *sclerotiorum* from that of the control. The mycelium-grown PDA control showed regular and homogenous hyphae (Fig 6A and 6B). The mycelium was also characterized by the morphology of uniseriate, uniform, and robust hyphae with constant diameter. However, shriveled hyphal aggregates and collapsed hyphae (Fig 6C and 6D) were observed after the application of essential oil. Significant changes also existed in sclerotial morphology (Fig 6E and 6F). Dill seed essential oil used at a concentration of 1.00 μL/mL (contact phase) caused disruption and loss of integrity in rind globular cells (arrow in Fig 6G), as well as deformation on rind globular cells inside the sclerotia (Fig 6G and 6H).

**Fig 7:**
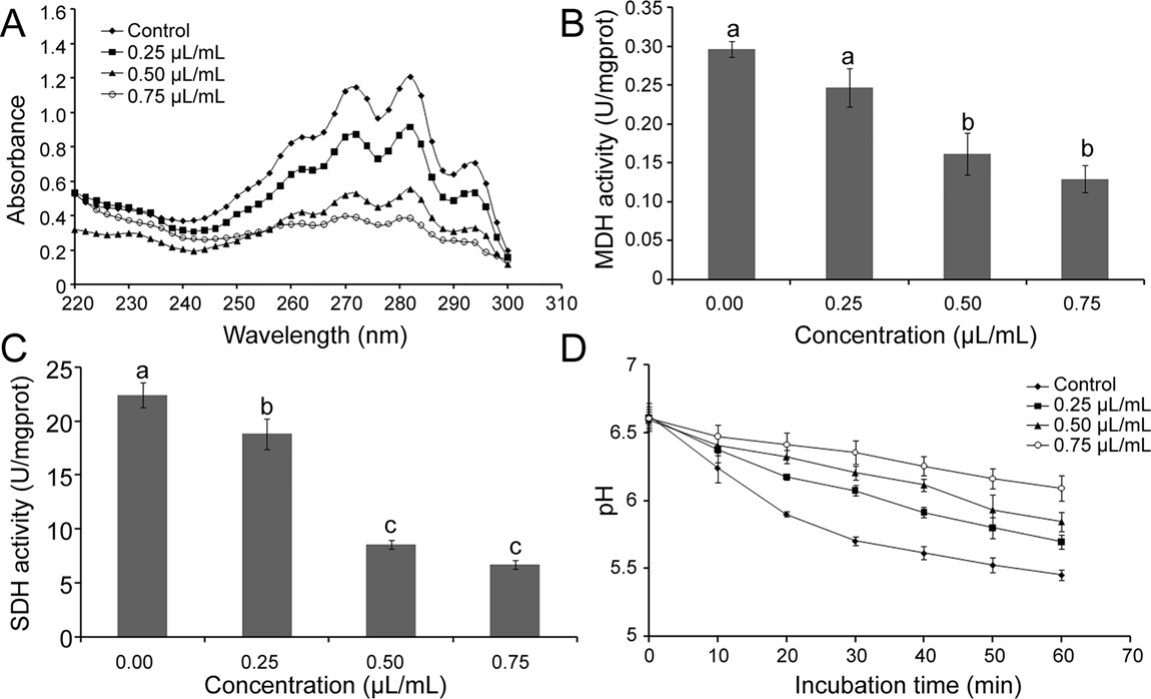
Some possible mechanisms of the oil against *S*. *sclerotiorum*. (A) UV spectrophotometric sterol profiles of *S*. *sclerotiorum* treated with the oil in comparison with those of the untreated control. (B) Effect of the oil on activity of malate dehydrogenase. (C) Effect of the oil on activity of succinate dehydrogenase. (D) Inhibitory effect of the oil on glucose-dependent acidification of medium in *S*. *sclerotiorum*. Significant differences (*p* < 0.05) between means are indicated by the letters above histogram bars. Values are means (*n* = 3) ± standard deviations.

The results of ergosterol production in the plasma membrane of *S*. *sclerotiorum* treated with different concentrations of dill seed essential oil are shown in Fig 7A. Absorbance at 282 nm was decreased with an increasing concentration of essential oil. The essential oil can significantly decrease the ergosterol content in the plasma membrane of *S*. *sclerotiorum* (*p* < 0.05). With the increasing concentration of essential oil, both activities of MDH and SDH were decreased significantly, particularly at the concentrations of 0.5 and 0.75 μL/mL (*p* < 0.05) (Fig 7B and 7C).

The essential oil of dill seed can reverse the reduction of external pH value induced by glucose (Fig 7D). The inhibitory activity was enhanced with increasing essential oil concentration and incubation time. The group treated with 0.75 μL/mL essential oil can reduce the pH value from 6.62 to 6.09 after 60 min incubation, whereas the pH value of the control group changed from 6.62 to 5.45.

## Discussion

Many people pursue enhanced quality of life as the society progresses. Synthetic chemical fungicides currently used are harmful to the public health. Hence, biocontrol agents and plant secondary metabolites, such as essential oils and other aromatic volatile products, have been extensively studied for their activities against phytopathogenic fungi [22]. In this study, dill seed essential oil may be a potential novel fungicide in crops.

GC-MS analysis of dill seed essential oil showed that the major compounds identified with GC were carvone (41.51%) and limonene (32.63%). Previous studies have shown that essential oils isolated from different parts and the storage duration of dill from different regions are characterized by high contents of carvone and limonene [9, 23, 24]. Lazutka et al. [23] reported the chemical composition of the essential oil of the dill collected in Lithuania. According to their study, the main constituents of the oil were carvone (58.11%) and limonene (37.12%). Bailer et al. [24] also reported that the dill seed essential oil compositions of a number of dill cultivars in Austria are characterized by the presence of carvone (10.7 mg g^−1^ to 13 mg g^−1^) and limonene (10.21 mg g^−1^ to 17.82 mg g^−1^). Dill seed essential oil from the Netherlands was reported to contain 19.9% limonene and 17.2% carvone [25]. Oil obtained from stored dill seed in Bulgaria was characterized by high carvone (50.1%) and limonene (44.1%) [9]. Similar results were also obtained for dill seed essential oil from Portugal, Iran, and Egypt [26–28]. These variations are attributed to environmental conditions such as climate, location, seasonal factors, and development stage [29]. Generally, the volatile compound from the plant is considered non-phytotoxic and potentially effective against various food-borne, human, and plant pathogenic fungi and pests [13, 30, 31].

*In vitro* anti-*S*. *sclerotiorum* activities of extracts and essential oils from different plant species were previously reported [31]. Several previous studies have also demonstrated the antimicrobial activity of dill seed essential oil [9, 10]. Results of the present study indicate that the vapor phase of dill seed essential oil shows greater inhibition than the contact phase to the pathogen *in vitro*. Many investigators have revealed that the antifungal activity results from a direct effect of essential oil vapors on fungal mycelium, and they postulated that the lipophilic nature of essential oils allowed easy absorption by fungal mycelia [32]. The current results showed that carvone may contribute more than limonene in the effect of the essential oil, as the antifungal activity of carvone is better than limonene. This phenomenon may be attributed to the carbonyl group on the p-menthane skeleton based on their structures. Carvone and limonene presented a synergistic effect toward the interferential effect, that is, the effect of the mixture of carvone and limonene was better than both carvone and limonene. Therefore, carvone and limonene are the main active constituents in dill seed essential oil.

Previous *in vivo* studies on antifungal activities of various crude extracts and essential oils have revealed varying degrees of antifungal effects on different plant pathogenic fungi [33]. Our current study showed similar results of interference of dill seed essential oil against *S*. *sclerotiorum*-induced pathological process under controlled conditions *in vivo*. Greater concentrations of plant essential oils were required in plant leaves than in laboratory media; this result may be ascribed to the better nutritional and moisture conditions in the leaves than those in the laboratory media, thereby rendering faster microorganism growth [34].

*In vitro* SEM analysis of the microstructure of *S*. *sclerotiorum* revealed some mechanisms of dill seed essential oil. After treatment with the essential oil, shriveled hyphal and lytic sclerotia can be observed. Similar results were found in previous studies presenting microstructures of fungal hyphal and sclerotia treated with essential oils [18]. These modifications may be caused by the prevention of enzyme synthesis and the breakage of the cell wall structure, which may influence the cytoplasmic content and integrity of hyphal and sclerotia, ultimately inducing mycelial death [35]. Many studies have suggested that compounds penetrate the cell and interfere with cellular metabolism. Other studies have proposed that these compounds disrupt the cellular membrane and react with the active sites of enzymes or act as H^+^ carriers, thereby depleting the adenosine triphosphate pool [36].

To elucidate the mechanism of dill seed essential oil, the plasma membrane and mitochondria were regarded as antifungal targets [37]. Ergosterol, as a predominant part of the cell membrane, serves as a bioregulator of membrane fluidity and asymmetry, as well as membrane integrity of a fungal cell [38]. Several previous studies have revealed that ergosterol content play an important role in evaluating the mechanisms of action [20]. The effect of the essential oil on the cell membrane of *S*. *sclerotiorum* was evaluated by measuring the content of ergosterol that is essential for fungal structure and cell growth [39]. In our experiment, absorbances between 230 and 300 nm were recorded. The content of ergosterol decreased with an increasing concentration of dill seed essential oil. Thus, the cell membrane may be a target of dill seed essential oil based on the effect on ergosterol content.

MDH and SDH were used to catalyze enzymes in ATP biosynthesis. MDH catalyzes a dehydrogenation reaction from malic acid to generate oxaloacetic acid and plays crucial roles in various cellular processes [40]. SDH is one of the hubs that connect oxidative phosphorylation and electron transport, as well as a source of electrons in the respiratory chain of various eukaryotic mitochondria and prokaryotic cells. MDH and SDH are indicators of mitochondrial enzymes, which can be used to evaluate the effects of TCA cycle. The TCA cycle is essential in the metabolic network in all oxidative organisms and provides precursors for anabolic processes that drive the generation of energy [41]. Our results indicated that the activities of MDH and SDH were significantly decreased by the essential oil treatments, and the TCA cycle in the mitochondria was influenced.

Acidification of extracellular compartments involves ATP-dependent proton pumps inserted in their limiting membranes [42]. Various metabolic functions may also be affected by acidification and eventually induce cell death [20]. We examined the possibility that the cell membrane limited this acidic extracellular resorbing compartment. The high concentrations of dill seed essential oil can effectively inhibit such acidification compared with the control group. Thus, energy generation and metabolism of *S*. *sclerotiorum* may be affected by the essential oil.

In conclusion, both *in vitro* and *in vivo* results indicate that the essential oil is a significant potential fungicide and can be applied to oil crops, particularly rapeseed. The use of dill seed essential oil can improve crop safety by eliminating fungal spread without detectable residues. The main constituents, specifically carvone, will also interfere with the pathological processes. The mechanisms of interference primarily involve action on the plasma membrane and mitochondria. Thus, the use of dill seed essential oil will be an economic application with comparable commercial significance and a natural fungicide in treatment of this plant pathogen that caused severe destruction to crops. Dill seed essential oil as a fungicide against other microbial phytopathogens causing severe destruction to crops should also be investigated. Further studies are necessary to analyze the mechanisms of the main components of dill seed essential oil.

## Supporting Information

S1 Fig Structure of carvone (A) and limonene (B). (TIF)

S2 Fig MS spectrogram of chemical compositions in dill seed essential oil. (DOCX)

S1 Table Results of the samples at contact phase on colony diameter growth of *Sclerotinia sclerotiorum*. (DOCX)

S2 Table Results of the samples at vapor phase on colony diameter growth of *Sclerotinia sclerotiorum*. (DOCX)

S3 Table Results of the samples at contact phase on sclerotia germination of *Sclerotinia sclerotiorum*. (A) Dill seed essential oil, (B) Mixture of carvone and limonene, (C) Limonene, (D) Carvone.
(DOCX)

S4 Table Results of the samples at vapor phase on sclerotia germination of *Sclerotinia sclerotiorum*. (A) Dill seed essential oil, (B) Mixture of carvone and limonene, (C) Limonene, (D) Carvone.
(DOCX)

S5 Table Results of dill seed essential oil against *Sclerotinia sclerotiorum* in detached oilseed rape (*Brassica napus* L.) leaves. (A)Before inoculation, (B)After inoculation.
(DOCX)

S6 Table Results of dill seed essential oil against *Sclerotinia sclerotiorum* in potted oilseed rape plants. (DOCX)

S7 Table UV spectrophotometric sterol profiles of *Sclerotinia sclerotiorum* treated with the oil in comparison with those of the untreated control. (DOCX)

S8 Table Results of dill seed essential oil on activity of malate dehydrogenase (A) and succinate dehydrogenase (B). (DOCX)

S9 Table The change of pH value after treating with different concentration dill seed essential oil. (DOCX)
