# Supplementary figures and images for: Interference and Mechanism of Dill Seed Essential Oil and Contribution of Carvone and Limonene in Preventing Sclerotinia Rot of Rapeseed
Source: PLoS One. 2015 Jul 2;10(7):e0131733. doi: 10.1371/journal.pone.0131733 (PMC4489822; doi:10.1371/journal.pone.0131733)

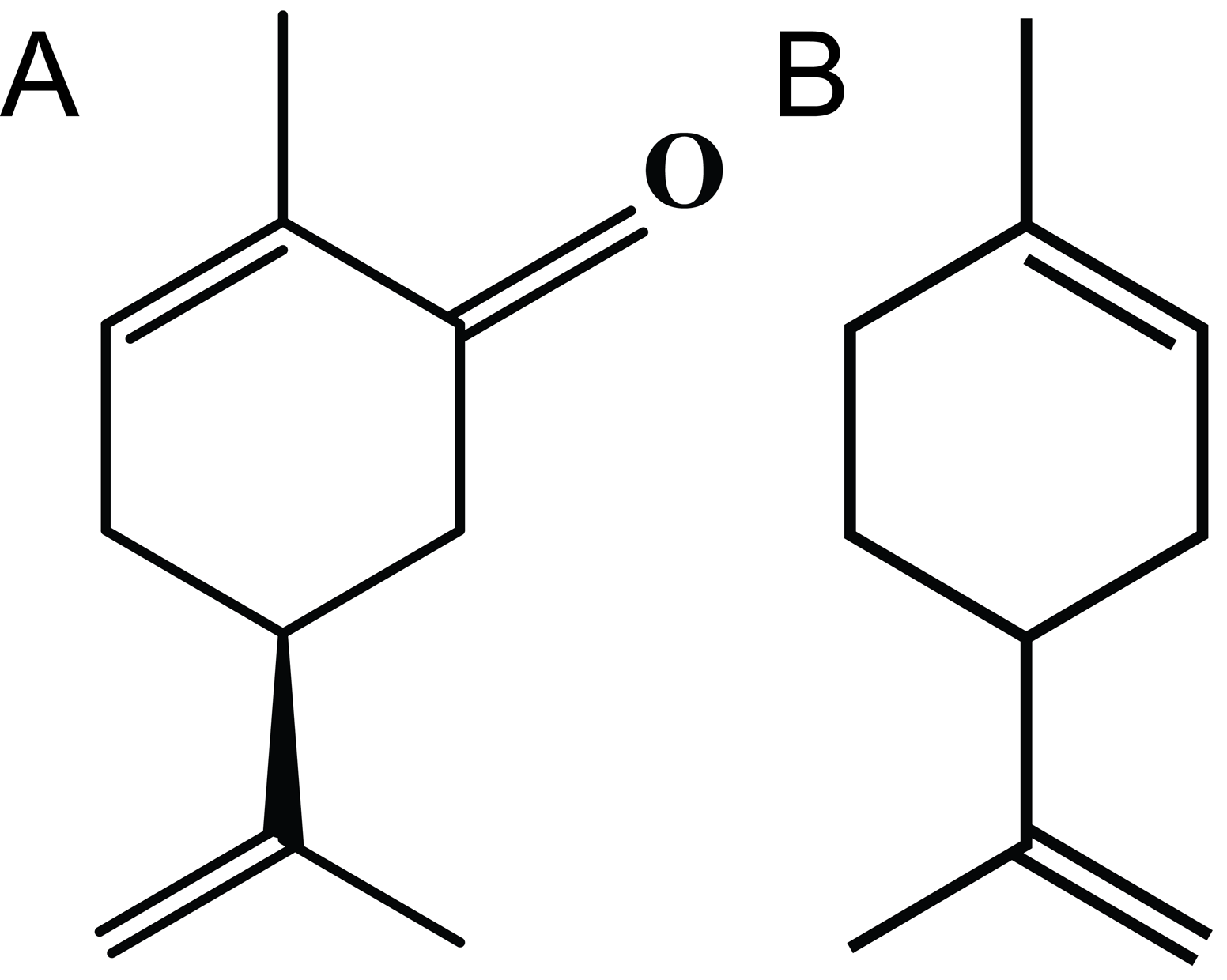

Supplement: S1 Fig — (TIF) [file pone.0131733.s001.tif]
