# Supplementary material for: Interference and Mechanism of Dill Seed Essential Oil and Contribution of Carvone and Limonene in Preventing Sclerotinia Rot of Rapeseed
Source: PLoS One. 2015 Jul 2;10(7):e0131733. doi: 10.1371/journal.pone.0131733 (PMC4489822; doi:10.1371/journal.pone.0131733)

S2 Fig. MS spectrogram of chemical compositions in dill seed essential oil

α- Thujene


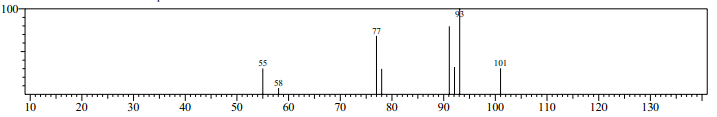


α- Pinene


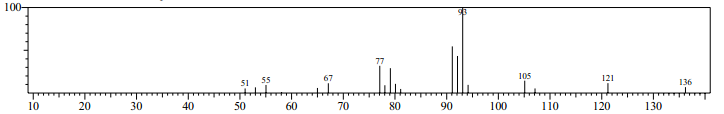


β- Myrcene


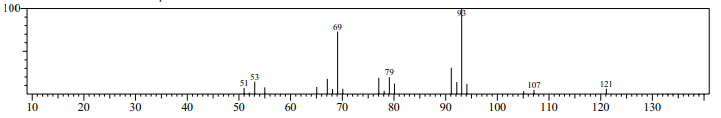


β- Phellandrene


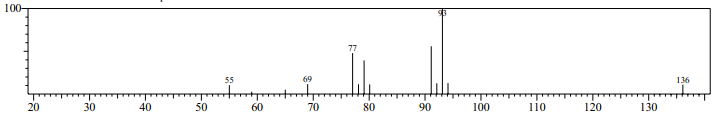


α- Phellandrene


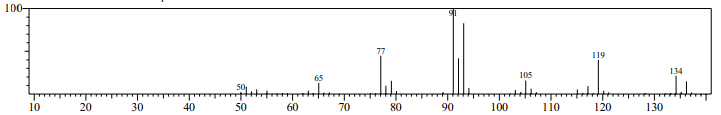


γ- Terpinene


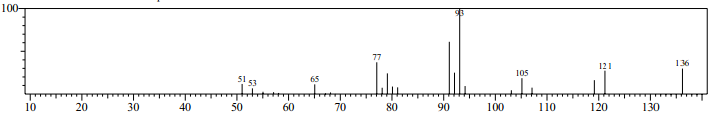


Limonene


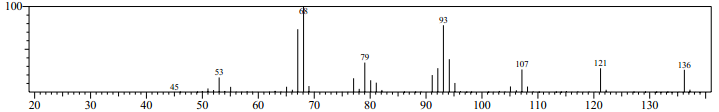


(*E*)- Limonene oxide


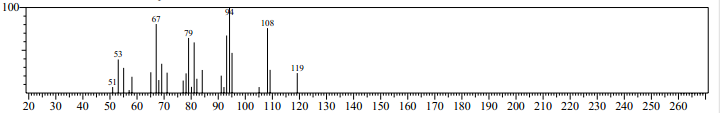


*p*-Cymene


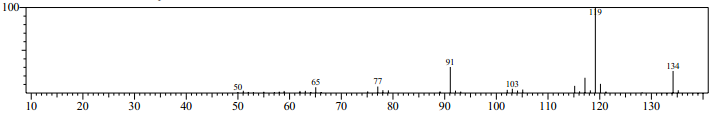


Anethofuran


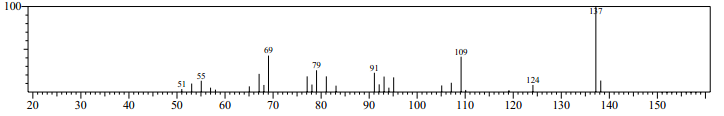


*trans*-Fenchone


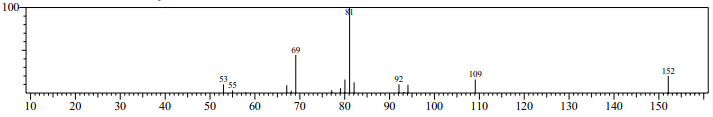


*cis*-Dihydrocarvone


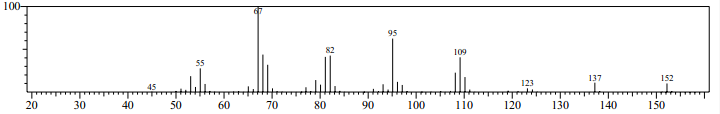


*trans*-Dihydrocarvone


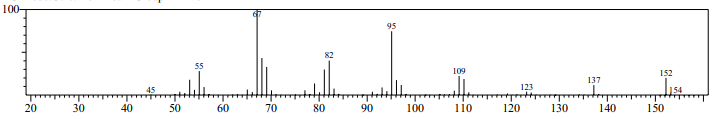


Carvone


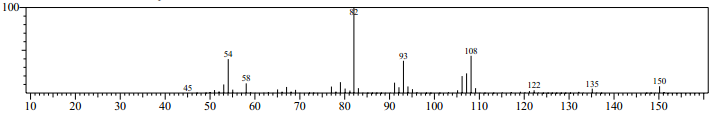


Dihydrocarveol


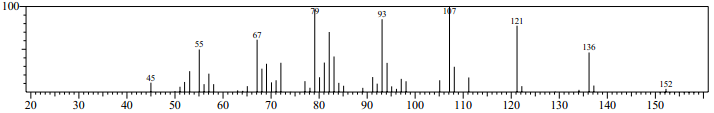


Neodihydrocarveol


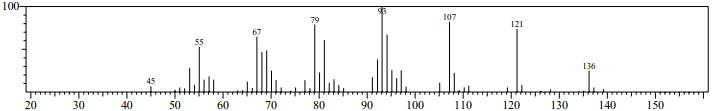


*cis*-Carveol


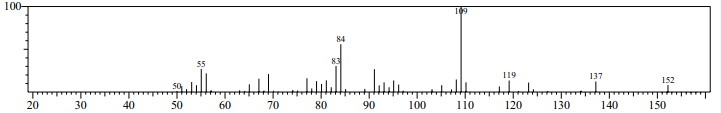


Caryophyllene oxide


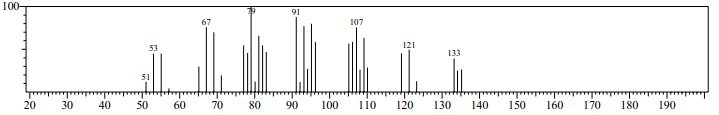


Myristicin


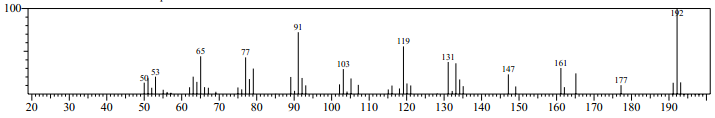


Asarone


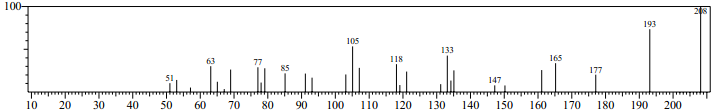


Apiol


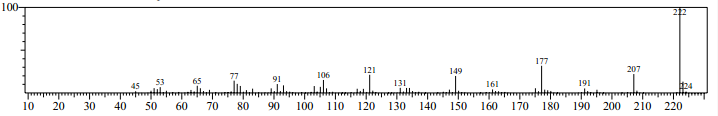

Supplement: S2 Fig — (DOCX) [file pone.0131733.s002.docx]
