# Supplementary material for: Interference and Mechanism of Dill Seed Essential Oil and Contribution of Carvone and Limonene in Preventing Sclerotinia Rot of Rapeseed
Source: PLoS One. 2015 Jul 2;10(7):e0131733. doi: 10.1371/journal.pone.0131733 (PMC4489822; doi:10.1371/journal.pone.0131733)
Supplement: S1 Table — (DOCX) [file pone.0131733.s003.docx]

S1 Table. Results of the samples at contact phase on colony diameter growth of *Sclerotinia sclerotiorum*.

|  | Colony diameter (cm) | | | | | | | | | | | | | | | | | | | |
| --- | --- | --- | --- | --- | --- | --- | --- | --- | --- | --- | --- | --- | --- | --- | --- | --- | --- | --- | --- | --- |
| Samples | Dill seed essential oil | | | | | Mixture of carvone and limonene | | | | | Limonene | | | | | Carvone | | | | |
| Concentration | 0 | 0.25 | 0.5 | 0.75 | 1 | 0 | 0.18 | 0.36 | 0.55 | 0.75 | 0 | 0.08 | 0.16 | 0.24 | 0.33 | 0 | 0.1 | 0.2 | 0.31 | 0.42 |
| (μl/ml) |  |  |  |  |  |  |  |  |  |  |  |  |  |  |  |  |  |  |  |  |
| D1 | 1.48 | 2.14 | 1.3 | 0.84 | 0.6 | 1.48 | 1.4 | 1.54 | 1 | 0.84 | 1.48 | 1.37 | 1.2 | 1.52 | 0.56 | 1.55 | 1.8 | 1.56 | 1.16 | 0.6 |
|  | 1.62 | 1.65 | 1.3 | 0.56 | 0.6 | 1.62 | 1.75 | 1.26 | 1 | 0.56 | 1.62 | 1.82 | 1.55 | 1.38 | 0.7 | 1.62 | 1.8 | 1.84 | 1.65 | 0.6 |
|  | 1.55 | 1.16 | 1.3 | 0.7 | 0.6 | 1.55 | 2.1 | 1.4 | 1 | 0.7 | 1.55 | 2.06 | 1.9 | 1.45 | 0.84 | 1.48 | 1.8 | 1.7 | 2.14 | 0.6 |
| D2 | 5 | 4.94 | 3.77 | 0.64 | 0.6 | 5 | 4.76 | 2.98 | 2.64 | 1.26 | 5 | 4.7 | 3.56 | 3.6 | 1.66 | 5 | 4.84 | 4.76 | 2.24 | 0.84 |
|  | 5 | 3.96 | 3.35 | 0.76 | 0.6 | 5 | 5.04 | 3.05 | 2.36 | 0.94 | 5 | 4.56 | 5.24 | 2.9 | 2.15 | 5 | 4.7 | 4.2 | 3.36 | 0.56 |
|  | 5 | 4.45 | 2.93 | 0.86 | 0.6 | 5 | 4.9 | 3.12 | 2.5 | 1.1 | 5 | 4.84 | 4.4 | 3.25 | 2.64 | 5 | 4.56 | 3.64 | 2.8 | 0.7 |
| D3 | 7 | 6.16 | 5.28 | 2.26 | 0.56 | 7 | 6.04 | 5.13 | 4.16 | 0.9 | 7 | 6.76 | 6.68 | 5.14 | 1.97 | 7 | 6.4 | 6.34 | 5.08 | 0.66 |
|  | 7 | 6.3 | 5 | 1.55 | 0.84 | 7 | 5.76 | 4.87 | 3.6 | 1.6 | 7 | 6.9 | 6.12 | 4.6 | 2.25 | 7 | 6.75 | 5.36 | 4.1 | 1.15 |
|  | 7 | 6.44 | 4.72 | 0.84 | 0.7 | 7 | 5.9 | 5 | 3.04 | 1.25 | 7 | 7.04 | 6.4 | 4.06 | 2.53 | 7 | 7.1 | 5.85 | 3.12 | 1.64 |
| D4 | 9 | 8.14 | 6.44 | 4.34 | 0.84 | 9 | 8.88 | 6.64 | 4 | 1.86 | 9 | 9 | 8.7 | 6.32 | 3.96 | 9 | 8.75 | 6.95 | 4.28 | 2.39 |
|  | 9 | 8.35 | 6.65 | 3.85 | 0.7 | 9 | 8.6 | 6.5 | 4 | 1.44 | 9 | 9 | 8.5 | 5.48 | 2.84 | 9 | 8.4 | 7.02 | 3.72 | 1.21 |
|  | 9 | 8.56 | 6.86 | 3.36 | 0.56 | 9 | 8.32 | 6.36 | 4 | 1.65 | 9 | 9 | 8.6 | 5.9 | 3.4 | 9 | 9.1 | 6.88 | 4 | 1.8 |
