# Supplementary material for: Interference and Mechanism of Dill Seed Essential Oil and Contribution of Carvone and Limonene in Preventing Sclerotinia Rot of Rapeseed
Source: PLoS One. 2015 Jul 2;10(7):e0131733. doi: 10.1371/journal.pone.0131733 (PMC4489822; doi:10.1371/journal.pone.0131733)
Supplement: S2 Table — (DOCX) [file pone.0131733.s004.docx]

S2 Table. Results of the samples at vapor phase on colony diameter growth of *Sclerotinia sclerotiorum*.

|  | Colony diameter (cm) | | | | | | | | | | | | | | | | | | | |
| --- | --- | --- | --- | --- | --- | --- | --- | --- | --- | --- | --- | --- | --- | --- | --- | --- | --- | --- | --- | --- |
| Samples | Dill seed essential oil | | | | | Mixture of carvone and limonene | | | | | Limonene | | | | | Carvone | | | | |
| Concentration | 0 | 0.05 | 0.075 | 0.1 | 0.125 | 0 | 0.037 | 0.055 | 0.073 | 0.093 | 0 | 0.016 | 0.024 | 0.033 | 0.041 | 0 | 0.021 | 0.031 | 0.042 | 0.052 |
| (μl/ml) |  |  |  |  |  |  |  |  |  |  |  |  |  |  |  |  |  |  |  |  |
| D1 | 1.52 | 0.6 | 0.6 | 0.6 | 0.6 | 1.52 | 0.6 | 0.6 | 0.6 | 0.6 | 1.52 | 0.6 | 0.6 | 0.6 | 0.6 | 1.52 | 0.6 | 0.6 | 0.6 | 0.6 |
|  | 1.5 | 0.6 | 0.6 | 0.6 | 0.6 | 1.5 | 0.6 | 0.6 | 0.6 | 0.6 | 1.5 | 0.6 | 0.6 | 0.6 | 0.6 | 1.5 | 0.6 | 0.6 | 0.6 | 0.6 |
|  | 1.63 | 0.6 | 0.6 | 0.6 | 0.6 | 1.63 | 0.6 | 0.6 | 0.6 | 0.6 | 1.63 | 0.6 | 0.6 | 0.6 | 0.6 | 1.63 | 0.6 | 0.6 | 0.6 | 0.6 |
| D2 | 5 | 1.2 | 0.6 | 0.6 | 0.6 | 5 | 0.96 | 0.6 | 0.6 | 0.6 | 5 | 1.15 | 1.04 | 0.84 | 0.6 | 5 | 1.08 | 0.6 | 0.6 | 0.6 |
|  | 5 | 0.8 | 0.6 | 0.6 | 0.6 | 5 | 1.24 | 0.6 | 0.6 | 0.6 | 5 | 1.35 | 1.15 | 0.66 | 0.6 | 5 | 0.85 | 0.6 | 0.6 | 0.6 |
|  | 5 | 1 | 0.6 | 0.6 | 0.6 | 5 | 1.1 | 0.6 | 0.6 | 0.6 | 5 | 1.25 | 0.95 | 0.75 | 0.6 | 5 | 1.24 | 0.6 | 0.6 | 0.6 |
| D3 | 7 | 1.5 | 1.05 | 0.6 | 0.6 | 7 | 1.16 | 1.04 | 0.6 | 0.6 | 7 | 4.04 | 3.6 | 1.96 | 1.36 | 7 | 1.52 | 1.34 | 0.6 | 0.6 |
|  | 7 | 2.5 | 0.75 | 0.6 | 0.6 | 7 | 1.44 | 0.76 | 0.6 | 0.6 | 7 | 3.76 | 2.9 | 2.24 | 0.94 | 7 | 1.38 | 1.06 | 0.6 | 0.6 |
|  | 7 | 2 | 0.9 | 0.6 | 0.6 | 7 | 1.3 | 0.9 | 0.6 | 0.6 | 7 | 3.9 | 3.25 | 2.1 | 1.15 | 7 | 1.45 | 1.2 | 0.6 | 0.6 |
| D4 | 9 | 3.6 | 1.56 | 1.04 | 0.6 | 9 | 3.6 | 1.86 | 0.6 | 0.6 | 9 | 7.05 | 4.24 | 3.56 | 3.12 | 9 | 4.12 | 2.52 | 0.97 | 0.6 |
|  | 9 | 2.9 | 1.14 | 0.76 | 0.6 | 9 | 2.9 | 1.44 | 0.6 | 0.6 | 9 | 5.45 | 3.96 | 3.14 | 2.98 | 9 | 3.98 | 2.38 | 0.98 | 0.6 |
|  | 9 | 3.25 | 1.35 | 0.9 | 0.6 | 9 | 3.25 | 1.65 | 0.6 | 0.6 | 9 | 6.25 | 4.1 | 3.35 | 3.05 | 9 | 4.05 | 2.45 | 1.05 | 0.6 |
