# Supplementary material for: Interference and Mechanism of Dill Seed Essential Oil and Contribution of Carvone and Limonene in Preventing Sclerotinia Rot of Rapeseed
Source: PLoS One. 2015 Jul 2;10(7):e0131733. doi: 10.1371/journal.pone.0131733 (PMC4489822; doi:10.1371/journal.pone.0131733)
Supplement: S4 Table — (A) Dill seed essential oil, (B) Mixture of carvone and limonene, (C) Limonene, (D) Carvone. (DOCX) [file pone.0131733.s006.docx]

S4 Table. Results of the samples at vapor phase on sclerotia germination of *Sclerotinia sclerotiorum*.

(A) Dill seed essential oil

| Concentration (μl/ml) | 0.000 | 0.050 | 0.075 | 0.100 | 0.125 |
| --- | --- | --- | --- | --- | --- |
| Sclerotia germination percent (%) | 100 | 100 | 70 | 50 | 0 |
|  | 100 | 90 | 80 | 40 | 0 |
|  | 100 | 90 | 70 | 40 | 0 |

(B) Mixture of carvone and limonene

| Concentration (μl/ml) | 0.000 | 0.037 | 0.055 | 0.073 | 0.093 |
| --- | --- | --- | --- | --- | --- |
| Sclerotia germination percent (%) | 100 | 90 | 70 | 50 | 0 |
|  | 100 | 100 | 80 | 50 | 0 |
|  | 100 | 90 | 70 | 50 | 0 |

(C) Limonene

| Concentration (μl/ml) | 0.000 | 0.016 | 0.024 | 0.033 | 0.041 |
| --- | --- | --- | --- | --- | --- |
| Sclerotia germination percent (%) | 100 | 100 | 100 | 80 | 30 |
|  | 100 | 100 | 90 | 70 | 30 |
|  | 100 | 100 | 90 | 80 | 30 |

(D) Carvone

| Concentration (μl/ml) | 0.000 | 0.021 | 0.031 | 0.042 | 0.052 |
| --- | --- | --- | --- | --- | --- |
| Sclerotia germination percent (%) | 100 | 100 | 80 | 60 | 10 |
|  | 100 | 90 | 80 | 40 | 0 |
|  | 100 | 90 | 70 | 50 | 10 |
