# Supplementary material for: Interference and Mechanism of Dill Seed Essential Oil and Contribution of Carvone and Limonene in Preventing Sclerotinia Rot of Rapeseed
Source: PLoS One. 2015 Jul 2;10(7):e0131733. doi: 10.1371/journal.pone.0131733 (PMC4489822; doi:10.1371/journal.pone.0131733)
Supplement: S5 Table — (A)Before inoculation, (B)After inoculation. (DOCX) [file pone.0131733.s007.docx]

S5 Table. Results of dill seed essential oil against *Sclerotinia sclerotiorum* in detached oilseed rape (*Brassica napus* L.) leaves

1. Before inoculation

| Material | Concentration | Lesion diameter(cm) | | | | | |
| --- | --- | --- | --- | --- | --- | --- | --- |
| Dill seed essential oil | 0.25μl/ml | 1.25 | 1.25 | 1.15 | 1.2 | 1.25 | 1.15 |
|  | 0.5μl/ml | 0 | 0 | 0 | 0 | 0 | 0 |
|  | 0.75μl/ml | 0 | 0 | 0 | 0 | 0 | 0 |
|  | 1μl/ml | 0 | 0 | 0 | 0 | 0 | 0 |
| Tween 20 | 0.10% | 3.05 | 2.9 | 3.25 | 2.8 | 3.25 | 2.9 |
| Carbendazol | 1.00 mg/ml | 3.2 | 2.95 | 2.8 | 2.65 | 2.8 | 2.65 |

1. After inoculation

| Material | Concentration | Lesion diameter(cm) | | | | | |
| --- | --- | --- | --- | --- | --- | --- | --- |
| Dill seed essential oil | 0.25μl/ml | 1.6 | 1.95 | 1.75 | 1.55 | 2.1 | 1.75 |
|  | 0.5μl/ml | 1.3 | 1.2 | 1.3 | 1.35 | 1.35 | 1.3 |
|  | 0.75μl/ml | 1.15 | 0.95 | 1 | 1.15 | 1.25 | 1.05 |
|  | 1μl/ml | 0.85 | 0.95 | 0.85 | 0.9 | 0.85 | 0.8 |
| Tween 20 | 0.10% | 3.3 | 3.15 | 3 | 3 | 2.8 | 3 |
| Carbendazol | 1.00 mg/ml | 2.9 | 2.85 | 2.6 | 2.85 | 2.3 | 2.5 |
