# Supplementary material for: Interference and Mechanism of Dill Seed Essential Oil and Contribution of Carvone and Limonene in Preventing Sclerotinia Rot of Rapeseed
Source: PLoS One. 2015 Jul 2;10(7):e0131733. doi: 10.1371/journal.pone.0131733 (PMC4489822; doi:10.1371/journal.pone.0131733)
Supplement: S6 Table — (DOCX) [file pone.0131733.s008.docx]

S6 Table. Results of dill seed essential oil against *Sclerotinia sclerotiorum* in potted oilseed rape plants

| Material | Samples | Lesion diameter (cm) | | | | | |
| --- | --- | --- | --- | --- | --- | --- | --- |
| Dill seed essential oil | 1.25μl/ml | 1.72 | 1.85 | 1.66 | 1.91 | 1.48 | 1.63 |
|  | 2.50μl/ml | 1.46 | 1.69 | 1.55 | 1.37 | 1.52 | 1.74 |
|  | 5.00μl/ml | 0.98 | 0.82 | 1.13 | 1.09 | 0.85 | 1.15 |
|  | 10.00μl/ml | 0 | 0 | 0 | 0 | 0 | 0 |
| Tween 20 | 0.10% | 2.85 | 3.07 | 2.96 | 3.11 | 2.74 | 2.58 |
| Carbendazol | 1.00 mg/ml | 1.35 | 1.16 | 1.24 | 0.93 | 1.2 | 1.03 |
