# Supplementary material for: Interference and Mechanism of Dill Seed Essential Oil and Contribution of Carvone and Limonene in Preventing Sclerotinia Rot of Rapeseed
Source: PLoS One. 2015 Jul 2;10(7):e0131733. doi: 10.1371/journal.pone.0131733 (PMC4489822; doi:10.1371/journal.pone.0131733)
Supplement: S7 Table — (DOCX) [file pone.0131733.s009.docx]

S7 Table. UV spectrophotometric sterol profiles of *Sclerotinia sclerotiorum* treated with the oil in comparison with those of the untreated control

| λ | Absorbance | | | |
| --- | --- | --- | --- | --- |
|  | Control | 0.25μl/ml | 0.5μl/ml | 0.75μl/ml |
| 300 | 0.194 | 0.159 | 0.107 | 0.093 |
| 298 | 0.354 | 0.278 | 0.177 | 0.128 |
| 296 | 0.592 | 0.452 | 0.278 | 0.18 |
| 294 | 0.704 | 0.534 | 0.324 | 0.206 |
| 292 | 0.685 | 0.52 | 0.317 | 0.203 |
| 290 | 0.639 | 0.488 | 0.299 | 0.196 |
| 288 | 0.667 | 0.509 | 0.312 | 0.203 |
| 286 | 0.831 | 0.628 | 0.383 | 0.239 |
| 284 | 1.078 | 0.81 | 0.489 | 0.293 |
| 282 | 1.204 | 0.904 | 0.543 | 0.322 |
| 280 | 1.138 | 0.857 | 0.515 | 0.31 |
| 278 | 1.019 | 0.772 | 0.465 | 0.287 |
| 276 | 0.967 | 0.736 | 0.445 | 0.278 |
| 274 | 1.046 | 0.794 | 0.48 | 0.297 |
| 272 | 1.147 | 0.869 | 0.524 | 0.32 |
| 270 | 1.128 | 0.855 | 0.517 | 0.32 |
| 268 | 1.006 | 0.768 | 0.467 | 0.298 |
| 266 | 0.896 | 0.688 | 0.422 | 0.278 |
| 264 | 0.858 | 0.661 | 0.409 | 0.276 |
| 262 | 0.863 | 0.666 | 0.414 | 0.282 |
| 260 | 0.829 | 0.642 | 0.401 | 0.28 |
| 258 | 0.739 | 0.578 | 0.363 | 0.265 |
| 256 | 0.648 | 0.511 | 0.324 | 0.246 |
| 254 | 0.592 | 0.468 | 0.298 | 0.232 |
| 252 | 0.559 | 0.442 | 0.28 | 0.222 |
| 250 | 0.513 | 0.405 | 0.257 | 0.209 |
| 248 | 0.456 | 0.361 | 0.23 | 0.194 |
| 246 | 0.41 | 0.328 | 0.21 | 0.183 |
| 244 | 0.387 | 0.313 | 0.201 | 0.176 |
| 242 | 0.376 | 0.309 | 0.199 | 0.182 |
| 240 | 0.369 | 0.312 | 0.205 | 0.191 |
| 238 | 0.376 | 0.329 | 0.222 | 0.211 |
| 236 | 0.396 | 0.364 | 0.248 | 0.237 |
| 234 | 0.415 | 0.401 | 0.269 | 0.259 |
| 232 | 0.424 | 0.42 | 0.28 | 0.271 |
| 230 | 0.429 | 0.446 | 0.285 | 0.277 |
| 228 | 0.433 | 0.458 | 0.286 | 0.277 |
| 226 | 0.438 | 0.465 | 0.286 | 0.278 |
| 224 | 0.45 | 0.478 | 0.287 | 0.28 |
| 222 | 0.478 | 0.5 | 0.296 | 0.289 |
| 220 | 0.52 | 0.53 | 0.31 | 0.302 |
