# Supplementary material for: Interference and Mechanism of Dill Seed Essential Oil and Contribution of Carvone and Limonene in Preventing Sclerotinia Rot of Rapeseed
Source: PLoS One. 2015 Jul 2;10(7):e0131733. doi: 10.1371/journal.pone.0131733 (PMC4489822; doi:10.1371/journal.pone.0131733)
Supplement: S8 Table — (DOCX) [file pone.0131733.s010.docx]

S8 Table. Results of dill seed essential oil on activity of malate dehydrogenase (A) and succinate dehydrogenase (B)

| Concentration (μl/ml) | Control | 0.25 | 0.5 | 0.75 |
| --- | --- | --- | --- | --- |
| MDH activity (U/mgprot) | 0.29 | 0.27 | 0.13 | 0.15 |
|  | 0.31 | 0.22 | 0.16 | 0.12 |
|  | 0.29 | 0.25 | 0.19 | 0.12 |

(B)

| Concentration (μl/ml) | Control | 0.25 | 0.5 | 0.75 |
| --- | --- | --- | --- | --- |
| SDH activity (U/mgprot) | 23.60 | 21.60 | 21.90 | 23.60 |
|  | 19.00 | 17.30 | 20.10 | 19.00 |
|  | 9.00 | 8.60 | 8.20 | 9.00 |
