# Supplementary material for: Interference and Mechanism of Dill Seed Essential Oil and Contribution of Carvone and Limonene in Preventing Sclerotinia Rot of Rapeseed
Source: PLoS One. 2015 Jul 2;10(7):e0131733. doi: 10.1371/journal.pone.0131733 (PMC4489822; doi:10.1371/journal.pone.0131733)
Supplement: S9 Table — (DOCX) [file pone.0131733.s011.docx]

S9 Table. The change of pH value after treating with different concentration dill seed essential oil

| Time  (min) | pH | | | | | | | | | | | |
| --- | --- | --- | --- | --- | --- | --- | --- | --- | --- | --- | --- | --- |
|  | Control | | | 0.25μl/ml | | | 0.5μl/ml | | | 0.75μl/ml | | |
| 0 | 6.53 | 6.71 | 6.63 | 6.7 | 6.57 | 6.55 | 6.52 | 6.68 | 6.57 | 6.62 | 6.64 | 6.55 |
| 10 | 6.13 | 6.24 | 6.35 | 6.46 | 6.39 | 6.27 | 6.47 | 6.41 | 6.33 | 6.57 | 6.43 | 6.41 |
| 20 | 5.92 | 5.9 | 5.88 | 6.15 | 6.18 | 6.19 | 6.32 | 6.37 | 6.27 | 6.51 | 6.38 | 6.34 |
| 30 | 5.68 | 5.74 | 5.69 | 6.08 | 6.03 | 6.11 | 6.21 | 6.25 | 6.15 | 6.45 | 6.32 | 6.29 |
| 40 | 5.56 | 5.63 | 5.65 | 5.92 | 5.87 | 5.95 | 6.16 | 6.11 | 6.07 | 6.33 | 6.24 | 6.19 |
| 50 | 5.47 | 5.58 | 5.53 | 5.84 | 5.71 | 5.85 | 6.04 | 5.93 | 5.82 | 6.24 | 6.15 | 6.09 |
| 60 | 5.41 | 5.48 | 5.47 | 5.75 | 5.69 | 5.65 | 5.91 | 5.85 | 5.77 | 6.19 | 6.07 | 6.01 |
